# Characterization and Evaluation of Novel Film Forming Polymer for Drug Delivery

**Published:** 2011

**Authors:** Artish Mundada, Prashant Satturwar, Suniket Fulzele, Sudhir Joshi, Avinash Dorle

**Affiliations:** a *Department of Pharmaceutical Sciences, R.T.M. Nagpur University Campus, Amravati road, Nagpur- 440 033. INDIA.*; b *Faculty of Pharmacy, University of Montreal, Centre-ville, Montreal, Canada, H3C 3J7.*; c *College of Pharmacy and Pharmaceutical Sciences, Florida A & M University, Tallahassee, FL 32301, USA.*

**Keywords:** Damar Batu, Pellets, Sustained release, Films, Protective coating

## Abstract

DB is a whitish to yellowish resin, characterized initially in terms of solubility, acid value, molecular weight (M_w_), polydispersity index (M_w_/M_n_) and glass transition temperature (T_g_). Neat plasticized films of DB (Damar Batu) are investigated for mechanical, water vapor transmission and moisture absorption properties. To improve the mechanical properties of the free films dibutyl sebacate, a hydrophobic plasticizer was added to film composition. The biomaterial was further investigated for sustaining the drug release from spherical units (multiparticulates). The core of pellet was prepared using Diclofenac sodium (10% w/w) as a model drug by extrusion and speronization. The drug containing pellets were coated using DB plasticized film-coating solutions.

With 2% coat build-up, sustained drug release up to 10 h was achieved with coating solution containing 20% and 30% w/w (based on DB weight) plasticizers. Less than 3% drug was released in the first 2 h which may be explained in terms of the insolubility of DB and the drug in acidic milieu. The release from pellets coated using DB film coating solution containing 20% and 30% plasticizers followed first order release pattern.

DB seems to be a promising film former for pharmaceutical coating due to its reasonably good mechanical properties, low water vapor transmission and sustained release capability.

## Introduction

Film coating is one of the most commonly used methods for the preparation of sustained and controlled release dosage forms (1) leading to the evaluation of various materials with film forming property. The drug delivery applications of polymer or biomaterial films are well established for providing protective coatings and controlled drug release from dosage forms (2). The main parameters used to characterize the film forming materials for coating purposes are mechanical properties (3, 4), permeability properties (5) and water vapor transmission rates (6). Coated formulations for drug release behavior have been investigated for different conditions by various investigators (7-9).

To modify the release of active substances, oral sustained and controlled release formulations are used. Multiple unit dosage forms have attracted much attention among the sustained release dosage forms. After the ingestion, multiple unit dosage forms readily divide into sustained release units throughout the gastrointestinal tract (GI tract). ‘Pellet’ is one of the popular multiple unit dosage forms which is suitable for combination of incompatible drugs or situations in which different release rates of drugs are needed from the same dosage forms, in addition to *in-vivo *advantage such as reduction in the risk of dose dumping, reduced local irritation and more predictable drug release profiles (10). Pellets prepared by coating process for sustained release, are composed of two important parts; the core of pellets and coated layer. Extrusion and spheronization is one of the most widely used pelletization processes in the pharmaceutical industry as the processing time required is short. After introducing the extruder and spheronizer, the pelletization process has changed noticeably. The pelletization process results in an improved granulation shape and narrow size distribution. The process of pelletization using extrusion and spheronization involves various stages like wet massing, extrusion, spheronization and dryness. Extrusion spheronization offers some advantages like spherical pellets produced with smooth surface, narrow particle size distribution and minimal friability with ability to incorporate high levels of active substances up to 60-80%. Despite an extensive research on cellulosic and acrylic polymers that have good film forming property, problems still arise when they are sprayed on the tablets or pellets surface (7). Tackiness, undesired agglomeration and cracking tendency in dry coating lead to wrong coating composition. Due to such limitations associated with the existing film formers, there is always a constant quest for novel polymers or biomaterials with better film forming characteristics.

Damars are solid resins, generally less hard and durable than the copals, and white to yellow in color. They are obtained from *Shorea *spp. Like *S. lamellata *Foxw., *S. virescens *Parijs, *S. retinodes *Sloot., *S. guiso *and *S. robusta, Dipterocarpaceae *Family (11). They are distinguished from copal by means of their solubility in hydrocarbon-type solvents and drying oils. DB contains about 40% α-resin, 22% *β*-resin, 23% dammarol acid and 2.5% water (12). DB is mainly used as an emulsifier and stabilizer for the production of color, paints, inks and aromatic emulsions in food and cosmetic industries and also in manufacture of paper, wood, varnishes, lacquers, polishes and additives for beverages (13, 14). It has been also tried as water resistant coating and in pharmaceutical and dental industries for its strong binding properties (15). It has also been tried in pharmaceutical as a matrix former to sustain the drug release (16). 

In the present study, our aim was to evaluate DB as a novel biomaterial for pharmaceutical coating. Non-plasticized and plasticized films of DB were studied for their mechanical, water vapor transmission and moisture absorption properties. The pellets coated with plasticized film forming solution of DB were investigated for drug release characteristics.

## Experimental

*Materials*

Damar Batu (R.R.Enterprises, Mumbai, India) Dibutyl Sebacate (DBS) was received from Morflex Inc., Greensboro, NC. Diclofenac Sodium was obtained as a gift sample from Zim Laboratories, Nagpur, India. All other chemicals were of analytical grade and purchased locally.

*Polymer characterization*

DB was purchased locally and initially characterized for various physicochemical properties like color, acid value, softening point and solubility (17). Acid value calculated by the formula, acid value = 5.61 n/w, where, n = number of mL of 0.1 M potassium hydroxide required and W = weight in grams of substance. The softening point determined by Herculus drop technique. For solubility, 6 g gum sample in 10 mL organic solvent and 3 g gum sample in 10 mL of different pH buffer was placed in a test tube mounted on water bath shaker for 24 h. Then, 2 mL of the mixture was transferred to a porcelain dish and the solvent was evaporated. Half of the weight gain of porcelain dish after complete solvent evaporation was taken as solubility. Molecular weight (M_w_) and glass transition temperature (T_g_) was determined using gel permeation chromatography and differential scanning calorimeter respectively. Polymer samples for molecular weight were eluted through a PL gel 3 μ mixed column at a flow rate of a 1.0 mL/min using tetrahydrofuran as a solvent using a gel permeation chromatography system (Perkin Elmer) equipped with a differential refractometer (La-Chom Detector L-7490). Polystyrene standards (polysciences) were used for calibration purpose. For the determination of the glass transition temperature approximately 6 mg of sample was placed on the aluminium pan and scanned over a temperature range of 25-250°C at a rate of 10°C /min using differential scanning calorimeter (DSC-Shimadzu 50). Samples were scanned in triplicate.

*Preparation of films and its characterization*

Films of DB were prepared on the mercury substrate by solvent casting method (18), using 20% w/v solution in Chloroform. To evaluate the plasticizer effect, plasticizer (Dibutyl sebacate) was added in concentration of 20% and 30% w/w and taken as the percentage of the total weight of polymer in solution. Casted films were dried at a room temperature for 24 h. The casted films after drying were carefully cut into film strips (Length: 42.4 mm; Width: 19.8 mm; Thickness: 0.8 mm) and investigated for the mechanical properties like tensile strength, percent elongation and young’s modulus (18) using Instron Instrument (model 4467, Instron Corp., Canton, MA) by ASTM standard test principle. The measurements were made at a crosshead speed of 5 mm/min and gauge length of 50 mm at 50% relative humidity (RH) and 23°C temperature. For each film specimen, all the parameters were determined in triplicate. Thickness gauge (Oswa scientific, Ambala, India) was used to determine film thickness (19) recorded in triplicate.

*Moisture absorption studies of films*

25 × 10 mm² strips of films were used for percent moisture absorption studies. Strips in tarred petri dishes were transferred to glass desiccators maintained at controlled relative humidities of 23, 43, 75 and 95% respectively. Different saturated solutions containing excess solute were used to control the relative humidity (RH) in the chamber. Accurately weighed film specimen were placed in various RH chambers and removed at the end of 14 days and weighed again. Percent moisture absorption is calculated using the following formula:

$\mathrm{Percent moisture absorption}=\frac{\mathrm{Final Wt.}-\mathrm{Initial Wt.}}{\mathrm{Initial Wt.}}\times 100$

*Water vapor transmission rate studies*

Water vapor transmission studies were done using the permeation cell consisted of a glass body (internal diameter = 2.25 cm; height = 8.0 cm) and a cup with an opening of 23.4 mm diameter (test area 4.17 cm²). The body and the cup of the cell were held in place with the help of three screw clamps. The polymeric films of the appropriate dimensions were cut and mounted on the permeation cell to determine the water vapor transmission rate (20, 21). To provide effective surface area for water vapor transmission, the under-investigation film was tightly clamped between the cup and the body of the permeation cell. The RH was maintained at 43% and 93% using saturated salt solution with excess solute of potassium carbonate and potassium nitrate respectively (22). The charged cells were weighed and transferred to the desiccators maintained at 0% RH. The cells were removed at the end of 24 h and reweighed. The amount of water vapor transmitted through the film was given in terms of the weight loss of the assembled cell. The Utsumi’s equation has been used to determine the water vapor transmission rate (23). Utsumi’s equation taking Film thickness into consideration is given as:

$Q=\frac{W\times L}{S}$

Where, W = Mass of water (g) transmitted per 24 h, L = Film thickness (cm), S = Surface area (cm²), Q = Water vapor transmission (g.cm/cm²)/24 h.

*Preparation and evaluation of diclofenac sodium pellets*

Pellet cores containing diclofenac sodium (DS 10% w/w) were prepared using extrusion- speronization. Microcrystalline cellulose (MCC) was chosen since it is commonly used as filler in aggregates applied in pharmaceutical production. These pellets generally showed a limited amount of fragmentation (24). DS and MCC (Avicel pH 101) were mixed and blended using binder solution to obtain wet mass that was transferred to a twin extruder and screened through 2 mm sieve at an extruder speed of 50 rpm. The extrudates so obtained were speronized at 1000 rpm for 5 min. The DS pellets were dried at 40°C for 6 h.

Physicochemical properties of DS pellet such as moisture absorption, particle size distribution, bulk density, flow rate, angle of repose and percent friability were evaluated (25). To determine the DS content in pellets, about 500 mg 14/20 mesh cut DS pellets (*i.e*. equivalent to 50 mg of DS) were transferred to 50 mL volumetric flask. Then, 40 mL methanol was added to the flask and the mixture was shaken continuously to extract drug completely. After that, volume of the solution was made to 50 mL with methanol. The mixture was filtered and the filtrate was collected. The content of DS in filtrate was determined using spectrophotometer (Shimadzu Inc., Japan) at 276 nm.

*Pharmaceutical coating*

The drug containing pellets were coated with plasticized DB film forming solution achieving 2% coat buildup without any significant agglomeration or tackiness. The coating of the pellets were done using spray rate of 1.0 mL/min, spray gun position 15 cm from pellet bed surface and atomizing pressure of 2.8 Kg/cm², under conditions of inlet air temperature of 70-75°C, with pellet bed temperature of 40-45°C.

*Scanning electron micrography (SEM)*

Scanning electron micrography (SEM) of whole intact pellets and cross-sections of pellets were done using scanning electron microscope (Stereoscan 250-MK-III). Samples for SEM of whole intact pellet and cross-sectioned pellet were prepared by splitting them with sharp blade and were fixed on spherical brass stub with the help of adhesive tape. Fixed samples were gold coated for 120 sec under Argon atmosphere using sputter coater before examination (26).

*In-vitro dissolution studies*

*In-vitro *drug release analysis was performed initially in 900 mL of 0.1 N HCl (pH 1.2) for 2 h and then in phosphate buffer pH 6.8 up to 10 h. Drug release from coated pellets was tested using USP XXIII dissolution apparatus 2 (Veego scientific, Mumbai, India) at 37°C at a speed of 50 rpm. Accurately weighed portion of about 500 mg of film coated DS pellets equivalent to 50 mg DS was added to each dissolution vessel. Aliquots of solution were withdrawn at predetermined time intervals and exchanged with new media of the same volume maintained at the same temperature. The amount of the drug released from the formulations was determined in each aliquot using spectrophotometer at 276 nm. The *in-vitro *dissolution studies were carried out in triplicate.

To determine the drug release kinetics, the *in-vitro *release data have been treated as each of the three kinetic models viz. Zero order release kinetics, First order release kinetics and Higuchi kinetics. 

## Results and Discussion

DB is a white to yellowish colored transparent crystalline material with softening point range of 90-93°C. The physicochemical properties of DB are shown in Table 1. DB has a narrow range of molecular weight distribution as indicated by the polydispersity index (M_w_/M_n_) of 1.7. The glass transition temperature of DB was found to be 38.79°C. Different solvents and different pH conditions were used to determine the solubility of DB. From the study, it was found that DB was practically insoluble in water and soluble in almost all organic solvents showing its hydrophobic nature. DB showed a pH dependent solubility as an acid resistant property (Table 2).

**Table 1:** Polymer characterization

**Parameters**	**DB**
Color	Grey brown
Acid value^*^	27.08
Softening point^*^	90-93°C
Mole.wt. (M_w_)	120
Polydispersity index (P.I. = M_w_/M_n_)	2
Glass transition temperature (T_g_)	38.79°C

^*^Represents average of five determinations

**Table 2:** Solubility data of DB showed a pH dependent solubility

**Solubility in different solvents**		**Solubility in different pH solutions**	
**Solvent**	**Solubility (g/mL)**	**pH**	**Solubility (g/mL)**
Chloroform	21.97 ± 0.010	1.2	21.0 ± 0.80 x 10^-2^
Dichloromethane	21.05 ± 0.020	4.6	35.2 ± 2.70 x 10^-2^
Acetone	0.40 ± 0.070	6.8	1.0 ± 2.7 x 10^-1^
Isopropyl alcohol	4.30 ± 0.050	8.0	80.5 ± 1.9 x 10^-2^
Ethanol	4.10 ± 0.013	10.0	255.6 ± 2.10 x 10^-2^
Water	Insoluble		

*Film characterization*

Non-plasticized DB films were smooth and transparent but slightly brittle and hence, the addition of plasticizer was found to be effective in improving the mechanical properties of free films. Plasticizer shifts the glass transition temperature to lower temperature and is an important formulation factor (27). Film characterization could not be carried out on non-plasticized films of DB, as they were brittle in the dried state. Due to the hydrophobic nature of DB, in this study DBS, which is hydrophobic plasticizer, has been utilized. Mechanical properties of the plasticized DB films containing 20% and 30% w/w DBS on the basis of total weight of polymer in solution, are shown in Table 3. The plasticized films with mean thickness of 45 to 70 mm showed moderate tensile strength and high elongation with sufficient flexibility to be bent in the dried state. The increase in elongation due to the addition of plasticizer may contribute in increased adhesion between film and coating surface. The results of mechanical property testing revealed that the plasticizer addition was effective for positively modifying the non-plasticized film characteristics of DB. Plasticized films were found to be moldable with sufficient flexibility.

**Table 3:** Mechanical properties of the plasticized films

**Material**	**Thickness of film (cm)**	**Tensile strength (MNm** ^-2^ **)**	**%Elongation**	**Young’s modulus (MNm** ^-2^ **)**
DB with 20% DBS	0.7	0.102	2.944	91.209
DB with 30% DBS	0.45	0.217	5.453	190.704

The results of water vapor transmission rate (WVTR) studies are shown in Table 4. Plasticizer addition was also found to affect the polymeric film permeability (7, 28). As the experimental humidity conditions affected the WVTR, humidity conditions of 43% and 93% were employed throughout the study. DB films demonstrated low WVTR indicative of its hydrophobic nature. As film thickness was likely to affect the WVTR, Utsumi’s equation used to determine the WVTR.

**Table 4:** WVTR of plasticized film of DB

**Material**	**Film thickness (cm)**	**Area (cm²)**	**WVTR (gm/cm²)/24 h at RH**	
			**43**	**93**
**DB with 20% DBS**	0.75(0.002)	4.17(0.19)	5.016 × 10^-3^	7.356 × 10^-3^
**DB with 30% DBS **	0.45(0.002)	4.17(0.19)	4.916 × 10^-3^	6.126 × 10^-3^

Mechanical properties were found to be considerably influenced by storage conditions along with polymer and type and also concentration of plasticizer, hence moisture absorption studies were carried out and the results are shown in Table 5.A and 5.B. Plasticized DB films showed the slight change in physical appearance.

**Table 5:** **(A) **Moisture absorption study of films of DB with 20 %w/w DBS as plasticizer

**RH%**	**Moisture absorbed%**
43	1.58
93	1.97
**(B) **Moisture absorption study of films of DB with 30% w/w DBS as plasticizer.	
**RH%**	**Moisture absorbed%**
43	0.66
93	4.28
**(C) **Moisture absorption study of uncoated pellets of DS.	
**RH%**	**Moisture absorbed%**
43	0.029
93	0.2607

*Characterization of uncoated DS pellets*

The physicochemical properties of uncoated pellets like moisture absorption (Table 5.C), sieve analysis, bulk density, tapped density, flow rate, angle of repose and Percent friability along with the drug content are depicted in Table 6. Results revealed that the DS pellets had narrow size distributions and the sieve fraction on the 14/20 mesh was about 84%. The pellets showed good flow rate and low percent friability (≤ 0.6%).

**Table 6:** Physicochemical properties of DS pellets

**Parameters**	**DS pellet**
Sieve% fraction on 14/20 mesh cut	84.00
Bulk density (gm/mL ± SD)	0.79 (0.02)
Tapped density (gm/mL ± SD)	0.81 (0.01)
Flow rate (gm/mL ± SD)	271.85 (7.37)
Angle of repose (^0^) ± SD	36.27 (1.04)
Friability (%)	0.60
Drug content (%)	99 ± 2

*Scanning electron micrography*

**Figure 1:**
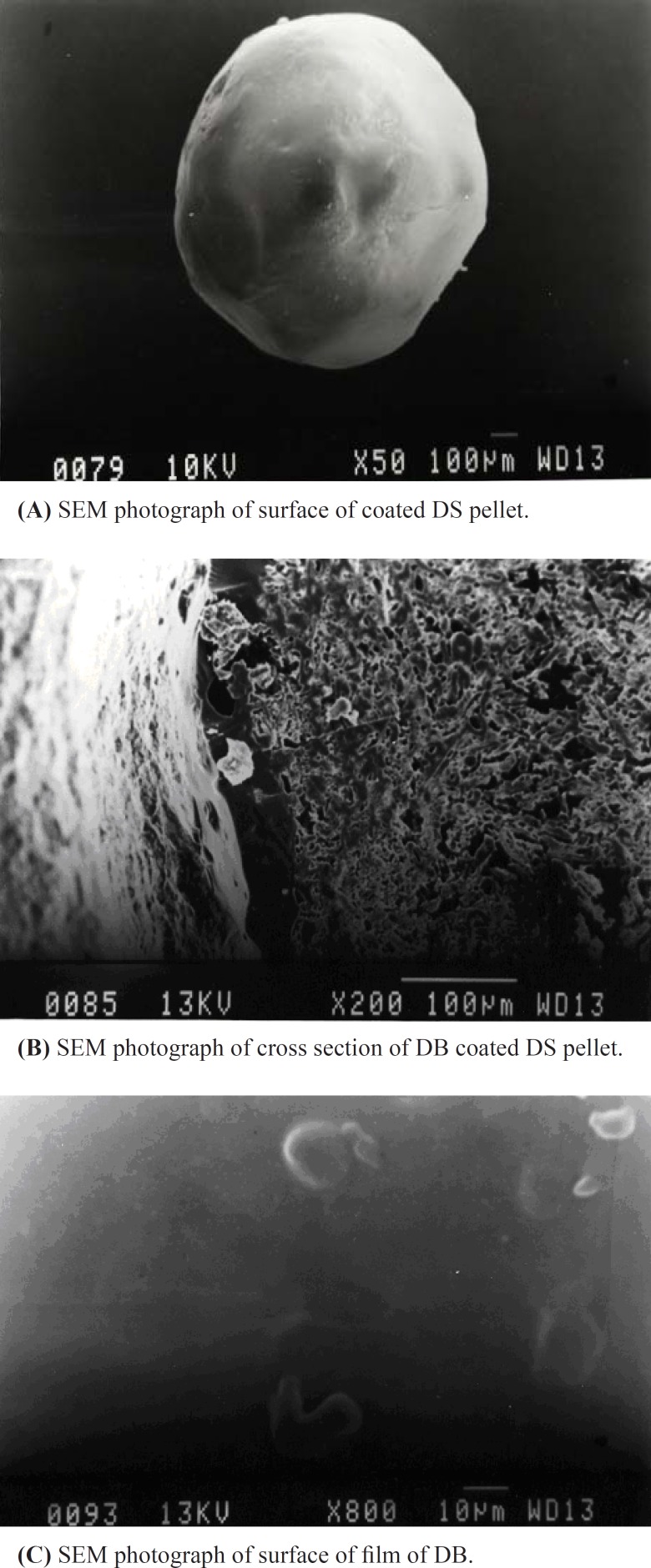
Surface and coating characterization of diclofenac sodium pellets made by scanning electron microscopy

Scanning electron microscopy studies were performed on coated DS pellet, cross section of coated DS pellet along with free film surface of DB. SEM views are shown in Figure 1.A, Figure 1.B and Figure 1.C respectively. SEM photographs illustrated that the coated pellet shows uniform and continuous appearance. Smooth and continuous surface of coated pellet may facilitate the sustained release of drug that is observed in the present study. Distinct layers of the coat and the core of the pellet is visible at higher magnification of the cross sectioned pellet which shows the efficiency of the coating. 

*In-vitro dissolution profile*

**Figure 2:**
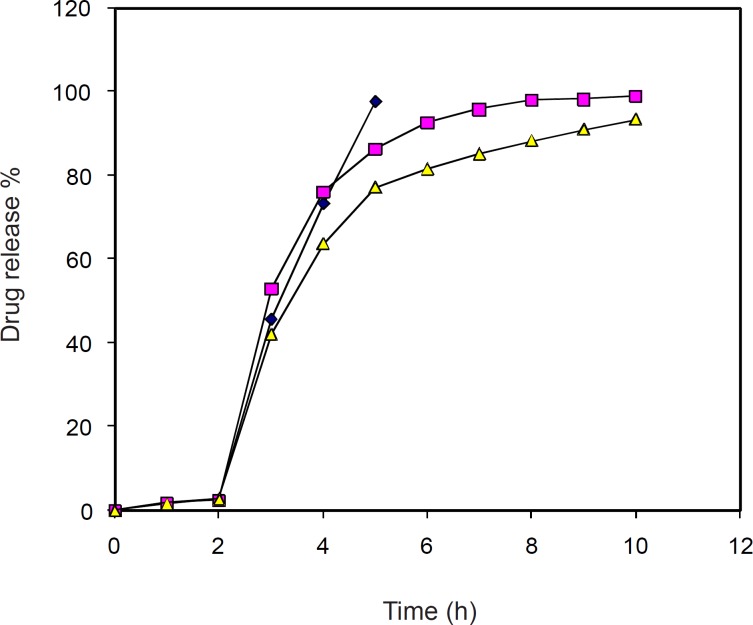
*In-vitro *release of diclofenac sodium from each formulation. Samples were withdrawn from dissolution apparatus at particular interval and analyzed by spectrophotometer for drug content. (A) Samples from uncoated formulation (♦). (B) Samples from formulation with 2% coat with 30% w/w plasticizer (■). (C) Samples from formulation with 2% coat with 20% w/w plasticizer (▲).

The dissolution profile of the uncoated and 2% coat buildup DS pellets with two different concentration of plasticizer is shown in Figure 2. Film coated DS pellets with plasticized DB coating solution had a round shape with fairly smooth surface before dissolution. After dissolution test, pellets still had a round shape but with little collapsed film and some pores on the film-coating layer. Very little amount of DS was released (< 3%) in first 2 h (pH = 1.2) which may possibly be due to the insolubility of DB and DS in acidic milieu, while at pH 6.8 release increased and subsequently sustained up to 10 h. 

Based on Table 7, it is clear that the release of drug from 2% coat build up pellets with 20% and 30% w/w plasticizer follows the first order release kinetics. 

**Table 7:** Regression coefficient values for zero order, first order and higuchi release kinetic models.

**(A) **For 2% coat build up with DB coating solution containing 30 % w/w DBS.		
**Zero order**	**First order**	**Higuchi kinetics**
0.8713	0.9789	0.9207
**(B) **For 2% coat build up with DB coating solution containing 20 % w/w DBS..		
**Zero order**	**First order**	**Higuchi kinetics**
0.9061	0.9889	0.9454

## Conclusions

A new pH dependent biomaterial DB with film forming property was examined for its drug delivery applications. Films prepared from novel biomaterial with 20% and 30% w/w plasticizer concentration on the basis of total weight of polymer in film forming solution were studied to determine the mechanical, WVTR and moisture absorption properties. Non-plasticized DB films were found to be slightly brittle and hence plasticizer was required to positively modify these films. DBS found to improve the film characteristics satisfactorily at the concentration of about 20% and 30% w/w. The release of DS from DB coated pellets was sustained up to 10 h. The release was found to be pH dependent and explained in terms of poor solubility of DB and DS in acidic milieu. From the data obtained from the present study, it may be proposed that DBS, which is a hydrophobic plasticizer, is effective in DB film formulations and such films can be used in pharmaceutical coating processes to design the sustained release dosage forms. Due to the low WVTR and sustained drug release capability, DB seems to be promising film forming polymer for pharmaceutical coatings.
